# Passion, Motivation, and Subjective Well-Being in Sport for People with Disabilities

**DOI:** 10.3390/healthcare13151919

**Published:** 2025-08-06

**Authors:** Luís Cid, Anabela Vitorino, Teresa Bento, Diogo Teixeira, Pedro Duarte-Mendes, Nuno Couto

**Affiliations:** 1 Sport Sciences School of Rio Maior, Santarém Polytechnic University (ESDRM-IPSantarém), 2040-413 Rio Maior, Portugal; luiscid@esdrm.ipsantarem.pt (L.C.); anabelav@esdrm.ipsantarem.pt (A.V.); teresabento@esdrm.ipsantarem.pt (T.B.); 2 Research Center in Sports Sciences, Health Sciences and Human Development (CIDESD), 5000-558 Vila Real, Portugal; 3 Faculty of Physical Education and Sport, Lusófona University, 1749-024 Lisbon, Portugal; diogo.teixeira@ulusofona.pt; 4 Research Center in Sport, Physical Education, and Exercise and Health (CIDEFES), 1749-024 Lisbon, Portugal; 5 Department of Sports and Well-Being, Polytechnic Institute of Castelo Branco, 6000-084 Castelo Branco, Portugal; pedromendes@ipcb.pt; 6 Sport Physical Activity and Health Research & Innovation Center (SPRINT), 6000-084 Castelo Branco, Portugal

**Keywords:** adapted sport, passion, behavioral regulation, satisfaction with life, affect, well-being

## Abstract

**Objective:** Considering the absence of research testing the entire sequence of passion, behavioral regulation, and subjective well-being (SWB), this study aimed to analyze the hypothetical causal relationships between the variables of a model that integrates the Dualistic Passion Model (DMP) and Self-Determination Theory (SDT) in order to understand the impact of harmonious passion (HP) and obsessive passion (OP) and the regulation of motivation on the SWB of elite athletes with disability. **Method:** This study includes 143 elite athletes from national adapted sports (36 female; 107 male) aged between 15 and 59 (M = 29.21; SD = 10.45). Weekly training sessions ranged from 1 to 12 (M = 4.52; SD = 2.71), and the years of competitive practice ranged from 1 to 28 (M = 5.55; SD = 6.98). Data were collected using valid and reliable questionnaires for the study population and analyzed using structural equation analysis. The following results were identified: a positive and a significant effect between OP and self-determination motivation (SDM); a positive but not a significant effect between OP and non-self-determination motivation (NSDM); a significant effect between SDM and SWL and SDM and positive affect (PA); and, finally, a positive but non-significant effect between SDM and negative affect (NA). In contrast, there is a positive and significant effect between HP and SDM; a negative and significant effect between HP and NSDM; a positive but non-significant effect between NSDM and SWL; a negative and significant effect between NSDM and PA; and, finally, a positive and significant effect between NSDM and NA. **Conclusions:** The perception of passion regarding sport can be a positive predictor of SDM, which, in turn, can influence levels of SWB, both from a cognitive point of view (SWL) and from an emotional point of view (PA). This reinforces the positive effect of the self-determination behavior in adapted sport on SWB and its contribution to health and quality of life in people with disabilities.

## 1. Introduction

Athletes with disabilities participating in adapted sports often encounter challenges related to physical limitations, psychological barriers, and contextual constraints [1,2], which can influence performance, motivation, and well-being [3]. Although these can negatively impact their sporting experience, research has shown that the involvement of these athletes in a sport context can enhance psychological growth, self-efficacy, and subjective well-being (SWB) [4,5], since participation in sports can foster a supportive environment for personal growth, positive emotional balance, and self-determination motivation [6,7].

Self-Determination Theory (SDT, Deci & Ryan [8]) describes self-determined behavior along a motivational continuum from amotivation (lack of intention) to more autonomous types. Less self-determined behaviors are external regulation (by external rewards) and introjected regulation (by internal forces, such as guilt). More self-determined behaviors are identified as regulation (personal significance), integrated regulation (by personal values), and intrinsic motivation (by inherent joy) [9]. The theory points to fulfilling basic psychological needs of autonomy, competence, and relatedness, as necessary for well-being, and enabling the internalization of behaviors, supporting self-determination [10,11]. In the context of disabilities, studies such as those by Martin [2] and Jaarsma et al. [12] have shown that satisfying the psychological needs proposed by SDT is particularly relevant since athletes with disabilities often face social and structural barriers that can undermine their autonomy and sense of competence. Thus, the motivational support perceived in the adapted sports environment can play a key role in the internalization of sports practice and, consequently, in the increase of SWB.

Additionally, passion, which, according to the dualistic model of passion (DMP) [13] is conceptualized as the energy that sustains an individual’s commitment and persistence in sports, and its forms, harmonious passion (HP) and obsessive passion (OP), impact behavioral regulation differently. According to Vallerand et al. [13], HP is associated with autonomous regulation, where individuals are involved in activities voluntarily due to the appreciation they have for the activity and not to strengthen their identity, and, on the other hand, OP is linked to a controlling regulation, where individuals engage compulsively in a sport, motivated by the desire for social acceptance or the enhancement of self-esteem, urging themselves internally to do so, and each type impacts well-being differently, since HP is positively linked to well-being, and OP is negatively associated to well-being [14].

Effectively, regarding SWB, which is considered a long-term state in the presence of positive affect (PA) and absence of negative affect (NA) (i.e., affective dimension) and satisfaction with life (SWL) (i.e., cognitive dimension) [15,16], research has identified a positive association between passion, behavioral regulation, and SWB. In a sports context, Bento et al. [17] recently identified through a systematic review that HP and the autonomous regulation of behavior are positively related to SWB, and OP and controlled regulation of behavior are negatively associated with SWB. However, the authors also identified that none of the studies included in their review analyzed the full sequence of the model between passion, behavioral regulation, and SWB.

Related to the adapted sports context, the literature has shown that more self-determined behavior is positively linked to better perception of well-being and quality of life [18,19]; more committed to continued participation [6,20]; and better performance and technical and tactical development [21]. Recently, Teixeira et al. [21] tested, through a double-serial mediation, the effects of motivation and affective activation in the relationship between both types of passion and SWL in athletes from adapted sports, identifying that positive indirect effects through self-determined motivation emerged in the harmonious and obsessive passion models with a distinct and positive effect related to SWL and the empirical evidence showing that passion and behavioral regulation have a positive impact on well-being in people with disabilities who are involved in adapted sports. However, this exploratory study with serial regression tested the direct and indirect effects between model variables, assessing the role of the mediation of the effect between behavioral regulation and SWL, raising a theoretical hypothesis that affects SWL, which was not verified. Considering the SWB model, both dimensions (i.e., affective and cognitive) represent the SWB of the subject and are not dependent. Recently, Jacinto et al. [22] also verified this assumption in an adapted sports context since no mediation role was found to affect the years of sports practice in adapted sports and SWL.

Accordingly, since disabled elite athletes also face challenges that have a negative impact on their sporting lives, as well as general well-being, and since a gap has been identified by Bento et al. [17], namely, that there is no research empirically investigating the entire chain of passion, motivational regulation, and SWB, the present study is set to fill that gap. For the first time, this study empirically tests an integrated theoretical model that combines the DMP and SDT amongst disabled elite athletes. It tests the intermediation of the six forms of motivational regulation proposed by SDT (from amotivation to intrinsic motivation) on the relationship between both obsessive and harmonious passion and the dimensions of SWB (i.e., life satisfaction and affect). This study is set out to fortify the theoretical generalization of the DMP and SDT across sports psychology, specifically applied to disabled elite athletes.

It is expected that HP will be associated with more self-determined forms of motivational regulation (such as integrated and intrinsic motivation), which, in turn, are positively related to SWL and PA. On the other hand, OP tends to be associated with less self-determined regulations (such as external and introjected regulation), which are linked to a negative effect and lower satisfaction. Although these mechanisms have already been partially verified in the general population [23], few studies have tested this full mediation in a population with disabilities. Moreover, the current evidence [22,24] suggests different indirect effects that warrant further investigation. Therefore, the present study aims to fill this gap by testing, for the first time, an integrated model that assesses the specific mediations of the six forms of regulation from SDT between the two types of passion and the components of SWB in elite disability athletes.

## 2. Materials and Methods

### 2.1. Participants

In total, 143 elite athletes of adapted sports were enrolled voluntarily in this study (36 females and 107 males) from different adapted sports modalities (wheelchair handball, athletics, wheelchair basketball, boccia, canoeing, cycling, horse riding, 7-a-side football, goalball, judo, Greco-Roman wrestling, swimming, precision orientation, tricycle, and rowing), with ages ranging from 15 to 59 (M = 29.21; SD = 10.45). The weekly training frequency of this sample ranged between 1 and 12 sessions (M = 4.52; SD = 2.71), and years of experience ranged between 1 and 28 years (M = 5.55; SD = 6.98). All participants were required to be registered with the International Paralympic Committee (IPC) and to have participated in national or international formal competitive sports practice over the last 6 months. Overall, participants presented motor, sensory (visual and hearing), and/or cerebral paralysis disabilities.

### 2.2. Instruments

The reduced Portuguese version of the Passion Scale (PS) (Cid et al. [25]) was used to assess both types of passion (i.e., harmonious and obsessive) according to DMP [13]. This version is made up of eight items (four per factor) answered on a Likert-type scale, with seven answer possibilities ranging from 1 (“totally disagree”) to 7 (“totally agree”).

The Portuguese version of the Behavioral Regulation Sport Questionnaire (BRSQ) (Monteiro et al. [26]) is made up of 24 items (six per factor) answered on a Likert scale, with seven possible answers ranging from 1 (“not true of me”) to 7 (“completely true of me”), and assesses the different types of behavioral regulations linked to SDT’s motivational continuum [10]. In the present study, according to suggestions from Pelletier and Sarrazin [27], there were two composite factors: Self-Determined Motivation, indicating more autonomous regulation of motivation (i.e., identified regulation; integrated regulation; intrinsic motivation), and Non-Self-Determined Motivation, indicating less self-determined regulation of motivation (i.e., amotivation; external regulation; introjected regulation). This procedure was applied to reduce the complexity of the models to be tested [28].

The Portuguese version of the Positive and Negative Affect Schedule (PANAS) (Antunes et al. [29]) was used to assess PA and NA and the emotional dimension related to SWB. This version of the questionnaire consists of 10 items (5 per factor) answered on a 5-point Likert scale, with 5 possible answers ranging between 1 (“none or very slightly”) and 5 (“extremely”), which are arranged in two factors, which represent positive affect and negative affect.

The Portuguese version of the Satisfaction with Life Scale (SWLS) (Antunes et al. [30]) was used to measure the global cognitive judgement of an individual’s life satisfaction. This scale consists of five items answered through a Likert scale ranging from 1 (“strongly disagree”) to 7 (“strongly agree”). The items were grouped into one factor defined as satisfaction with life, which reflects the cognitive perspective of SWB identified by Diener et al. [15].

Although all instruments used in this study are validated in Portuguese and have demonstrated good internal consistency (α > 0.80), it is important to note that no specific validation was conducted for populations with disabilities. Nevertheless, previous studies [22,24] have employed these same instruments in similar contexts involving athletes with disabilities, supporting their appropriateness. In addition, care was taken to ensure comprehension and accessibility during data collection, as detailed below.

### 2.3. Data Collection

For this study, ethical approval was obtained from the Research Centre. The survey was completed by the disabled athletes, who were supported by trainers, family, or researchers, if assistance was needed, at the training site, internships, or during competitions. Athletes who needed support received help from trained researchers, coaches, or family members, ensuring that comprehension and autonomy were preserved as much as possible. The confidentiality and anonymity of the data collected were guaranteed, and all procedures were completed in accordance with the Helsinki Declaration [31]. Informed consent was individually obtained from all participants included in the study. The time required to complete the surveys ranged from 15 to 45 min.

### 2.4. Statistical Analysis

Descriptive statistics, including mean and standard deviation, as well as bivariate analysis, were conducted for all study variables. A two-step maximum likelihood (ML) approach was used, following Kline’s [32] recommendations. Firstly, a confirmatory factor analysis was performed to demonstrate the psychometric properties of the model. Composite reliability of each factor was calculated through the Raykov [33] formula, and average variance extracted was conducted to assess the convergent validity of the factors, as suggested by Hair et al. [34]. Discriminant validity was demonstrated when the average variance extracted for each construct exceeded the squared correlations between that construct and any other [34,35]. Secondly, a structural equation modeling approach was performed to check the associations across the variables under analysis. For both analysis, recommendations from several authors (e.g., Byrne [36]; Hair et al., [34]; Marsh, Hau, & When [37]) were followed, with traditional absolute and incremental indexes: standardized root mean square residual (SRMR), root mean square error of approximation (RMSEA) and its confidence interval (90% CI), comparative fit index (CFI), and non-normed fit index (NNFI), respectively. For these indexes, the cutoff values suggested by several authors (e.g., Byrne [36]; Hair et al., [34]; Marsh, Hau, & When [37]) were adopted, namely, SRMR ≤ 0.08, CFI, NNFI ≥ 0.90 and RMSEA ≤ 0.08. The analysis was undertaken using AMOS 23.0 software.

### 2.5. Mediation Analysis

Additionally, multiple mediation procedures were developed following Preacher and Hayes’ [38] and Hayes’ [39] recommendations. According to these authors’ suggestions, the PROCESS v. 3.1 macro for SPSS v27.0 was used, and model 4 with a six-parallel mediator was analyzed. This procedure allows the estimation of the direct and indirect effects in the proposed models while controlling the influence of k mediators between variables [39]. In the dependent–independent variables interaction, bias-corrected bootstrapped point estimates were calculated (considering standard errors and 95% CI). Significant indirect effects were considered present if the confidence interval did not include zero (at alpha = 0.005), using 5000 samples bootstrapping and bias-corrected and accelerated intervals to make inferences. As suggested by Hayes [39] and MacKinnon et al. [40], bootstrapping procedures are recommended as more efficient and powerful than normal theory approaches for detecting indirect effects in smaller samples.

## 3. Results

A preliminary inspection of the data revealed no violations of the univariate normal distribution, since the Kurtosis and asymmetry values were between −2 and 2 and −7 and 7 [34]. However, Mardia’s coefficient (110.93) was greater than the recommended value (5.0) suggested by Byrne [41]. Thus, following Nevitt and Hancock’s [42] recommendations, bootstrap Bollen–Stine (2000 samples) was used. There were also no multicollinearity problems, since the relationships between variables are all less than 0.90 [34].

In Table 1, the results regarding the descriptive, correlational analysis and composite reliability of the constructs of the hypothesized model are shown. The results reveal a significant correlation between all constructs in the analysis, except for correlations between HP-NA, OP-NSDM, OP-NA, SDM-NSDM, SDM-NA, NSDM-SWS, and PA-NA. It was also observed that the athletes value the constructs underlying HP, SDM, SWL, and PA in comparison to OP, NSDM, and NA. Finally, it was also observed that all factors have adjusted values of internal consistency and composite reliability > 0.70 [34], and there were no problems with convergent validity (AVE ≥ 0.50). Furthermore, no issues were identified with convergent validity (AVE ≥ 0.50) or discriminant validity, since the square of the correlations between the factors was lower than the AVE value of each of the factors [34].

With regards to model fitting (structural and measurement), all models presented satisfactory fit indexes according to the cutoff values fixed in the method (Table 2) [34,36,37].

**Figure 1:**
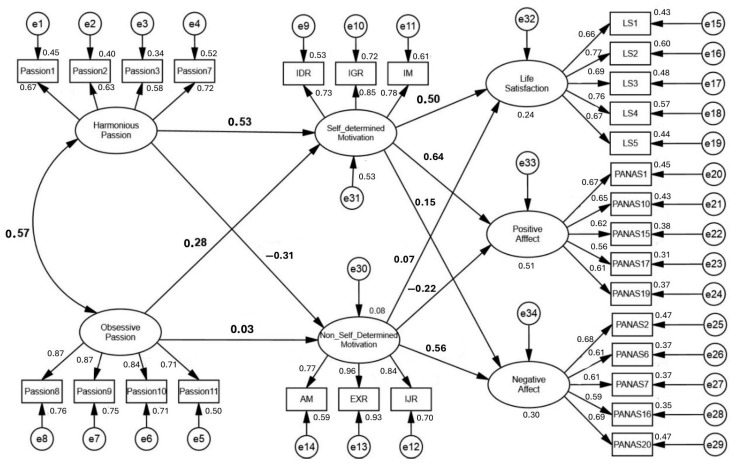
Individual parameters standardized from the model. **Note:** AM = amotivation; EXR = external regulation; IJR = introjected regulation; IDR = identified regulation; IGR = integrated regulation; IM = intrinsic motivation.

Figure 1 also presents a positive and significant association between HP and OP (r = 0.57). OP positively and significantly affected SDM (β = 0.28; 90% CI = 0.07 to 0.50, *p* = 0.02) but did not significantly affect NSDM (β = 0.03; 90% CI = −0.18 to 0.25, *p* = 0.77). SDM, in turn, presented positive and significant effects on SWL (β = 0.50; 90% CI = 0.36 to 0.63, *p* < 0.01; R^2^ = 0.24) and PA (β = 0.64; 90% CI = 0.48 to 0.77, *p* < 0.01; R^2^ = 0.51), but there was no significant effect on NA (β = 0.15; 90% CI = −0.02 to 0.31, *p* = 0.15). Positive and significant effects were also present between HP and SDM (β = 0.53; 90% CI = 0.28 to 0.73, *p* < 0.01; R^2^ = 0.53) and between HP and NSDM, but they were of a negative and significant nature (β = −0.31; 90% CI = −0.50 to −0.12, *p* = 0.01; R^2^ = 0.08). NSDM did not have a significant effect on SWL (β = 0.07; 90% CI = −0.10 to 0.22, *p* = 0.46), had a negative and significant effect on PA (β = −0.22; 90% CI = −0.39 to −0.07, *p* = 0.02), and had a positive and significant impact on NA (β = 0.56; 90% CI = 0.34 to 0.74, *p* = 0.001; R^2^ = 0.30).

Concerning standardized indirect effects, OP had significant and positive impacts on SWL (β = 0.14; 90% CI = 0.04 to 0.28, *p* = 0.01) and PA (β = 0.17; 90% CI = 0.04 to 0.33, *p* = 0.03) but not on NA (β = 0.06; 90% CI = −0.06 to 0.23, *p* = 0.45). HP also had significant indirect effects on SWL (β = 0.24; 90% CI = 0.10 to 0.39, *p* = 0.004) and PA (β = 0.41; 90% CI = 0.26 to 0.56, *p* = 0.01) but not on NA (β = −0.09; 90% CI = −0.26 to 0.06, *p* = 0.29).

**Figure 2:**
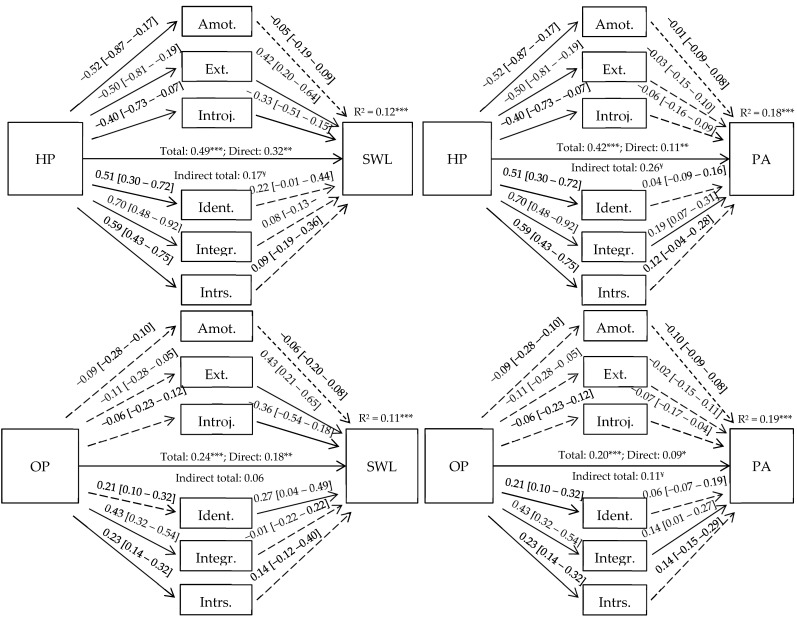
Direct and indirect effect analysis of motivational regulation in the relationship between the Dualistic Passion Model variables, SWL, and affect. Note. HP = harmonious passion; OP = obsessive passion; SWL = Satisfaction With Life; PA = positive affect; Amot. = amotivation; Ext. = external regulation; Introj. = introjected regulation; Ident. = identified regulation; Integr. = integrated regulation; Intrs. = intrinsic motivation; * *p* < 0.05; ** *p* < 0.01; *** *p* < 0.001; ¥: the 95% CI of the bias and corrected and accelerated estimate indicate a significant indirect effect; full arrow = significant effect, dashed arrow = non-significant effect.

In the mediation analysis (Figure 2), HP and OP had significant direct and/or total indirect effects on SWL and PA but not on NA (all *p*-values > 0.05). HP had total, direct, and indirect significant effects on SWL. Significant indirect effects were through external regulation (β = 0.42) and introjected regulation (β = −0.33) for the SWL model and through integrated regulation (β = 0.19) for the PA model. The same pattern also occurred for the OP models. For the SWL model, there were significant total and direct effects. Though there were significant indirect effects for a few specific paths of mediation (i.e., external, introjected, and identified regulation), there was no significant total indirect effect. For the PA model, there were significant total, direct, and total indirect effects, where integrated regulation presented an influential mediating effect (β = 0.14).

These results show that different forms of passion impact SWL through different processes of motivational regulation, and HP is more strongly connected to adaptive and self-determined processes.

## 4. Discussion

This study explores a structural model integrating the DMP, SDT, and SWB. The associations among these measures, as reported, correspond to previous research [43,44,45] and provide additional empirical support for the theoretical postulations that HP and autonomous motivation are positively correlated with athletes’ well-being. The ordering of associations verifies that athletes internalizing their motivational dispositions and engaging in sports for personal value reasons exhibit more positive affective outcomes and higher satisfaction with life. These findings are consonant with Vallerand’s [45] postulations, as well as the SDT model [46,47,48,49,50], providing support for the psychological benefit of self-determined motivational regulation in adapted sports environments. Lastly, the fit of the measurement model also reflects the strength of the constructs being measured, corresponding to previous validation research.

Secondly, it can be observed that all measurement models used were adjusted to the sample in question according to the cutoff values adopted in the methodology. Likewise, the values found in the measurement models are in line with studies of their respective validations. In addition, the theoretically recommended measurement model was adjusted to the data without presenting problems of discriminant or convergent validity. Finally, it is verified that all the constructs used present adequate internal consistency values, since the composite reliability values are all equal to or greater than 0.70 [34].

Regarding the hypothetical causal relationships established in the structural model (Figure 1) from the results obtained, it appears that the model, overall, adjusted to the data according to the cutoff values adopted in the methodology [34,37,41], partially confirming the established theoretical relationships (Figure 2).

The results demonstrate a positive and significant relationship between OP and HP. Such results are in line with previous studies, where the authors also found a positive relationship between these two types of passion (e.g., [44]), which reveals that subjects can simultaneously feel both types of passion in a specific activity, since the constructs are orthogonal [14,51].

The results show that HP and OP are positive and significant predictors of SDM and negative and significant predictors of NSDM. Although there appears to be no study that has empirically analyzed the relationships between types of passion and the regulation of motivation, the results obtained are in line with the theoretical relationships established in some studies (e.g., [14,45,52]. In fact, SDM is an extension of SDT because depending on the way in which the subject develops their passion for their sport, it can be internalized and integrated in a more autonomous/more self-determined or a more controlled/less self-determined way [10,14,51]. In other words, if the athlete has a harmonious passion for their sport, this is in line with other values in their lives; however, if the athlete has a more obsessive passion for their sport, their lives do not make sense without it, that is, it is the sport that controls the other domains of their lives (e.g., family, work, leisure, sleep, etc.) [14].

Furthermore, as mentioned in the previous paragraph, there seems to be no studies that empirically analyze the relationships between the two types of passion and the regulation of motivation. However, it was performed in another population (of non-disabled athletes). The work of Verner-Filion et al. [53] provides theoretical support for the positive contribution of HP to the prediction of BPN satisfaction, a pattern replicated in the present study among athletes in adapted sport. In this sense, if HP is a positive predictor of BPN, then it can be stated that the relationship found between HP and SDM is empirically accepted, since, according to SDT, for athletes to reach high levels of self-determined motivation, they need to satisfy their BPN of autonomy, competence, and relatedness, and this theoretical relationship has been demonstrated by research [43,49,50,51].

Although theoretical relationships suggest that OP has a negative relationship with SMD, this was not empirically verified in this study. The result obtained is not necessarily negative, given that an athlete can demonstrate OP for their sport and regulate their motivation in an autonomous/more self-determined way. This demonstration is because OP develops in an athlete’s perception of social acceptance and self-esteem, and even feelings of excitement arising from the activity, becoming dependent on the sport itself [54]. Still, if we look at the specific context where this work was developed, perhaps this relationship is natural, as people with disabilities can use sports to highlight and develop skills that are difficult to achieve in other social contexts [55]. In many cases, they seek in sports the visibility and social acceptance that they cannot obtain in other areas of experience and, with this, achieve higher levels of self-esteem and satisfaction with life.

According to Martin and Wheeler [56], these types of situations are used by individuals to develop their own resilience mechanisms, as they are not well accepted in other areas of society, and thus, through the regular practice of sports, they have the possibility of enhancing their abilities, skills, and personal resources, creating more effective and efficient coping strategies.

Broadly speaking, the higher levels of OP in athletes with a disability can be understood in the special significance of adapted sports to their lives. For the majority, sport ceases to be solely a competition: it becomes a unifying force, a source of personal development, and of rebuilding an identity. Participation in adapted sports has been linked to higher self-esteem, increased psychosocial health, and a sense of mastery and control, amounting to an athlete’s identity in place of disability-based stigmatizing labeling [54,55,56,57,58,59]

These results gain even more robustness since there is a negative and significant effect between HP and NSDM and a positive and non-significant effect between OP and NSDM. This evidence reveals that regardless of the type of passion felt, it is not important for NSDM. This fact corroborates the qualitative study [58] carried out with elite adapted sport athletes, demonstrating that sports participation was associated with the constructs of SDM. As already mentioned, DMP is an extension of SDT, more specifically, the theory of organismic integration (OIT), and this meta-theory explores social contexts, understanding how they promote or inhibit internalization and integration in behavior regulation [9]. If the OIT explores social contexts (where the athlete’s passion for their sport is found), then internalization and integration are processes that allow extrinsically motivated behaviors to become more self-determined. In other words, the OIT analyses explain how external regulations are incorporated into self-determination, based on a process of internalization that allows the subject to modify the conditions in which they are inserted [59], as recently demonstrated in a meta-analysis [60]. This internalization process is a guiding vehicle that allows athletes to reorganize behaviors that are extrinsically regulated to become more self-determined [10]. This means that, regardless of the type of passion felt by the athlete, it can influence SDM more than NSDM.

Regarding the relationships observed between SDM and SWB variables, there is a positive and significant effect between SDM and SWL and between SDM and positive affect but a non-significant effect between SDM and negative affect. These results are entirely justified from a theoretical point of view, as according to Ryan and Deci [9], high levels of autonomous motivation are associated with high levels of SWB, that is, when athletes regulate their behavior autonomously (i.e., identified regulation, integrated regulation, and intrinsic motivation), they will be closer to reaching high levels of well-being. Also, from an empirical point of view, these relationships are fully justified. For example, Núñez et al. [61], in a study carried out with 399 Spanish athletes across different sports, demonstrated that intrinsic motivation (one of the types of autonomous motivation) was a positive and significant predictor of psychological well-being. Likewise, Stenling et al. [62], in a longitudinal study (six months) carried out with Swedish athletes, demonstrated a positive and significant effect between the self-determination index and well-being.

Regarding the relationship found between SDM and NA, although positive, it was not significant, which means that it is not important for the model, that is, it is not valued by the athletes in this sample [41].

Finally, in relation to the relationships verified between NSDM and SWB, there is a positive, but not significant, effect between NSDM and SWL; a negative and significant effect between NSDM; and a positive and significant effect between NSDM and negative affect. These results are echoed theoretically, since when subjects regulate their behavior through controlled forms of motivation (i.e., amotivation, external, and introjected regulation), they experience more negative emotional and behavioral consequences, as seen in the present study in the relationship between positive affect and SWL [9].

Based on the results obtained in the direct and indirect path analysis of the hypothesized model, some authors [34] suggest that this may be an indicator of possible mediation interactions. According to these suggestions and several theoretical assumptions made throughout this work, the tested parallel mediation models reinforce the results trend, where both HP and OP present direct and significant effects on SWL and positive affect (but not NA), which is in line with previous study findings [14,44].

The indirect effects analysis suggests that motivational regulations partially mediate these relationships, particularly in the positive effect models, where the indirect total effect is always higher than the direct effect, corroborating SDT and DMP theoretical assumptions. The HP and OP paths to motivational regulations seem to follow the literature assumptions, presenting negative regression interactions with NSDM and positive regression interactions with SDM. In turn, all regulations (except external regulation) present the same expected trend interaction with SWL and positive affect. However, motivational regulations seem to partially mediate these relationships in a different manner. In SWL models, the NSDM appears to have some indirect effect contribution to the relationships studied, but in the positive effect models, it is the integrated regulation that emerges as the main significant indirect effect, justifying the mediator’s interactions. Particularly in the SWL models, external regulation seems to account for part of SWL, which may reflect, in these adapted sports participants, that some external others (e.g., family, coaches, and friends) may voluntarily or involuntarily impose some pressure for sports practice [14]. Additionally, the HP model distinguishes itself from the OP model in these mediation analyses in the magnitude of influences in SDM and NSDM mediators, where HP seems to better reflect SDT organismic integration assumptions and aligns perfectly with theoretical assumptions [13]. Thus, these results appear to suggest that these athletes, independently of the type of passion demonstrated, obtain different contributions from SDT motivational regulations to the studied outcome (SWL vs. positive affect).

Both these variables are not context-specific, meaning that the hypothesized model (and parallel mediation models) may suggest a natural contamination effect from sports practice to other life domains, which in the present study presents good indicators of well-being.

However, in the literature, there appears to be no study in this context that has analyzed the relationships between controlled motivation and well-being, as most studies focus on analyzing BPN [63] and autonomous motivation (or some form of autonomous motivation) [64] and their effects on well-being.

## 5. Practical Implications

The results of the current study have important practical implications. Here, the model was tested, indicating that OP and HP are positive predictors of self-determined/autonomous motivation and negative predictors of controlled/less self-determined motivation. Moreover, self-determined motivation positively predicts SWB, particularly PA and SWL, but controlled motivation is a negative predictor of such SWB measures.

The emerging sense of motivation that people with disabilities feel toward sports could play an appropriate role in fostering more self-determination-grounded motivation, which is also associated with higher SWB (cognitive-dimension SWL, as well as emotional-dimension PA). Such a motivational profile could also serve to expand persistence, long-term sport participation, and reduce sport dropout, all of which could enhance sport as an entry point for fostering health, quality of life, and social inclusion for people impacted by disability.

However, for these benefits to materialize as realities of practice, there is a necessity for sport program officials, sports psychologists, and coaches to adopt an evidence-based practice of promoting athletes with disabilities to have SDM and HP. It is achievable for coaches to support developing the formation of HP, facilitated by supporting athletes’ autonomy, as well as an expression of intrinsic value for sports practice as being something significant that is integrated into their sense of self. This involves setting personal goals for athletes, recognizing their personal achievements, and making engagement an expression of free choice as opposed to external coercion. Settings that facilitate basic psychological needs, competence, relatedness, and autonomy must be designed to sustain adaptive motivation. To maximize autonomy, athletes must be involved in decision-making regarding training as well as competitions. Competence can be enhanced by adequately demanding tasks and constructive and detailed performance feedback. Relatedness can be facilitated by group processes that facilitate teamwork, building rapport between teammates, and forming mutual support.

Support programs involving motivational interviewing, workshops for setting goals, and reflective exercises that allow athletes to connect sports participation with goals and values can also enhance motivation. Training programs must include SDT content so that experts can gain tools for identifying diverse forms of motivation and modulate their management and communication strategies accordingly to facilitate an inclusive and supportive motivational climate.

Adopting these practices can benefit not only peak performance and the health of disabled athletes but also create more accessible, inclusive, and sustainable sports environments. Particularly for the world of Paralympic and Deaflympic sports, where long-term engagement is strongest, these strategies could have a profound impact on athlete retention, as well as promoting their overall health.

## 6. Limitations and Agenda for the Future

Although the present study contributes to the adapted sport literature on psychological variables, there are also limitations.

Firstly, the cross-sectional design precludes inferring causality. Although there were correlational associations between passion, basic psychological needs, motivational regulation, and well-being, there is no ground from which the directions of these associations or their timings can be determined. This restricts the explanatory power of the findings, alongside the confidence that can underlie intervention design premised upon them. Only longitudinal research, conducted across time (e.g., across sports seasons or what would normally represent a Paralympic or Deaflympic cycle), can establish causal relationships and explore, across time, the dynamic development of these psychological processes, the interactions between them, and the potential antecedent changes they induce for well-being and performance outcomes. Experimental longitudinal trials, particularly those carried out within ecologically embedded real-world sport programs, would be particularly critical for inferring causality and ecological validity across a series of sport contexts and disability conditions.

Second, we employed composite measures of motivational regulation (i.e., self-determined/autonomous versus less self-determined/controlled motivation), which, however parsimonious and fitting as they are to the continuum framework proposed by SDT (e.g., through measures like the Relative Autonomy Index), can indirectly suppress the unique effects of each type of regulation (e.g., introjected, identified, and intrinsic regulations). For example, even if intrinsic and identified regulations are self-determined, they can have different influences on well-being. This is most problematic for complex populations like Paralympic or Deaflympic athletes, whose lives and mental repertoire may require more delicate tests. Future research would do well to look into each type of regulation individually to better discern subtle patterns of motivation and their unique contributions to psychological functioning, as well as to well-being.

Third, future studies could explore models of mediation, most prominent of which is the mediating role of basic psychological needs between the two forms of passion and motivational regulation, as well as between motivational regulation and basic psychological needs and the consequential outcomes of well-being. Such explorations would provide further understanding of the mechanisms underlying athlete functioning and flourishing.

Fourth, although the sample can also be considered representative of the study population, the sample is rather small (*n* = 143). This limits statistical power, as well as the generalization of findings. Larger samples would, for future work, better accommodate tests of more complex models, as well as comparisons of subgroups.

In addition, the broad age range of participants (15 to 59 years) may have contributed to additional variation in psychological responses. Age has also demonstrated an impact on motivational orientations, needs fulfilment, and perceptions of well-being. Although subgroup analyses (e.g., age, gender, sport, and disability type) would have resulted in a more complete analysis of the effects, they did not lie within the original parameters of the study and, therefore, were not conducted in order to prevent post hoc interpretations. Still, we recognize this as a limitation. Future studies would be aided by stratification **to** distinguish the joint operation of age-related distinctions from motivational/emotional processes within adapted sport.

## 7. Conclusions

In the absence of knowledge about these relationships, in the context of adapted sports (passion, regulation of motivation, and SWB), this work intends to contribute to their enrichment, especially in such a particular context. This fact reinforces the need for more studies in the context of adapted sports to understand how certain variables operate and, therefore, establish more effective and adjusted strategies, specifically in adapted competitive sports, as is the case with the sample in this research.

Despite the limitations, the results allow us to draw important lessons for practice, since the tested model highlights that both OP and HP are positive predictors of autonomous/self-determined motivation and negative predictors of controlled/less self-determined motivation. Furthermore, autonomous/self-determined motivation positively predicts well-being, especially positive affect and SWL, and controlled/less self-determined motivation negatively predicts well-being, PA, and NA. In other words, the fact that athletes with disabilities feel passionate about practising their sport can be a positive predictor of SDM, which, in turn, can influence levels of SWB, both from a cognitive point of view (SWL) and an emotional point of view (PA), thus providing an increase in persistence and continuation of the practice. It can contribute to reducing dropout rates in the adapted sports field and reinforce the role of sport as an essential strategy for promoting health, quality of life, SWL, and PA in people with disabilities.

## Figures and Tables

**Table 1:** Descriptive, correlational analysis, and composite reliability of the constructs of the hypothesized model.

Constructs	HP	OP	SDM	NSDM	SWL	PA	NA
HP	1	0.55 **	0.52 **	−0.26 **	0.35 **	0.45 **	0.02
OP	-	1	0.48 **	−0.09	0.33 **	0.42 **	0.09
SDM	-	-	1	−0.04	0.41 **	0.51 **	0.11
NSDM	-	-	-	1	−0.01	−0.24 **	0.47 **
SWL	-	-	-	-	1	0.25 **	−0.01
PA	-	-	-	-	-	1	−0.01
NA	-	-	-	-	-	-	1
Mean	6.19	5.05	5.64	2.61	5.20	4.02	1.70
SD	0.70	1.32	0.80	1.27	0.98	0.59	0.67
Min–Max	4–7	1–7	3–7	1–7	3–7	3–5	1–5
CR	0.73	0.89	0.81	0.87	0.83	0.73	0.80
AVE	0.52	0.68	0.63	0.74	0.51	0.53	0.52

Note: HP = harmonious passion; OP = obsessive passion; SDM = self-determination motivation; NSDM = non-self-determination motivation; SWL = satisfaction with life; PA = positive affect; NA = negative affect; SD = standard deviation; Min–Max = minimum–maximum; CR = composite reliability; AVE = average variance extracted; ** *p* < 0.01.

**Table 2:** Adjustment indexes of the tested models.

Model	χ^2^	df	B-S p	CFI	NNFI	SRMR	RMSEA	RMSEA-90%
PS	25.27	19	0.15	0.04	0.98	0.99	0.05	0.00–0.09
BRSQ	589.67	237	<0.01	0.91	0.90	0.06	0.08	0.08–0.09
SWLS	5.65	4	0.23	0.99	0.98	0.02	0.05	0.00–0.15
PANAS	58.85	32	0.01	0.90	0.93	0.07	0.08	0.08–0.09
Measurement Model	529.59	356	<0.01	0.90	0.90	0.08	0.06	0.05–0.07
Hypothesized Model	475.52	406	<0.01	0.93	0.92	0.08	0.05	0.03–0.06

Note. B-S p= bootstrap Bollen–Stine; df = degrees of freedom; p = significance level; CFI = comparative fit index; NNFI = non-normed fit index; SRMR = standardized root mean square residual; RMSEA = root mean square error of approximation; RMSEA-90% = root mean square error of approximation and confidence interval; PS = passion scale; BRSQ = Behavioral Regulation Sport Questionnaire; SWLS = Satisfaction with Life Scale; PANAS = Positive and Negative Affect Schedule.

## Data Availability

Data are available upon request to the corresponding author.
